# Concurrent chemoradiotherapy in adjuvant treatment of breast cancer

**DOI:** 10.1186/1748-717X-4-12

**Published:** 2009-04-07

**Authors:** Nabil Ismaili, Nawfel Mellas, Ouafae Masbah, Sanaa Elmajjaoui, Samia Arifi, Imane Bekkouch, Samir Ahid, Zakaria Bazid, Mohammed Adnane Tazi, Abdelouahed Erraki, Omar El Mesbahi, Noureddine Benjaafar, Brahim El Khalil El Gueddari, Mohammed Ismaili, Said Afqir, Hassan Errihani

**Affiliations:** 1 Department of Medical Oncology, National Institute of Oncology, Rabat, Morocco; 2 Department of Radiotherapy, National Institute of Oncology, Rabat, Morocco; 3 Department of Medical Oncology, Hassan II Hospital, Fes, Morocco; 4 Pharmacology and Toxicology Department, Faculty of Medicine, Rabat, Morocco; 5 Department of Medical Statistics, Faculty of Medicine, Rabat, Morocco; 6 Department of Cardiology B, Ibn-Sina Hospital, Rabat, Morocco; 7 Epidemiology Unit, National Institute of Oncology, Rabat, Morocco; 8 Department of Microbiology, Moulay Ismail University, Meknes, Morocco; 9 Department of Medical Oncology, Mohammed I Hospital, Oujda, Morocco

## Abstract

**Background:**

The optimal sequencing of chemotherapy and radiotherapy after breast surgery was largely studied but remains controversial. Concurrent chemo-radiotherapy is a valuable method for adjuvant treatment of breast cancer which is under ongoing research program in our hospital. We are evaluating the feasibility of the concomitant use of chemotherapy retrospectively.

**Methods:**

Two hundred forty four women having breast cancer were investigated in a retrospective study. All patients were either treated by radical surgery or breast conservative surgery. The study compares two adjuvant treatments associating concomitant chemotherapy and radiotherapy. In the first group (group A) the patients were treated by chemotherapy and radiotherapy in concomitant way using anthracycline (n = 110). In the second group (group B) the patients were treated by chemotherapy and radiotherapy in concomitant way using CMF treatment (n = 134). Chemotherapy was administered in six cycles, one each 3 weeks. Radiotherapy delivered a radiation dose of 50 Gy on the whole breast (or on the external wall) and/or on the lymphatic region. The Kaplan-Meier method was used to estimate the rates of disease free survival, loco-regional recurrence-free survival and overall survival. The Pearson Khi^2 ^test was used to analyse the homogeneity between the two groups. The log-rank test was used to evaluate the differences between the two groups A and B.

**Results:**

After 76.4 months median follow-up (65.3 months mean follow up), only one patient relapsed to loco-regional breast cancer when the treatment was based on anthracycline. However, 8 patients relapsed to loco-regional breast cancer when the treatment was based on CMF. In the anthracycline group, the disease free survival after 5 years, was 80.4% compared to 76.4% in the CMF group (Log-rank test: p = 0.136). The overall survival after 5 years was 82.5% and 81.1% in the anthracycline and CMF groups respectively (Log-rank test: p = 0.428). The loco-regional free survival at 5 years was equal to 98.6% in group A and 94% in group B (Log-rank test: p = 0,033). The rate of grade II and grade III anaemia was 13.9% and 6.7% in anthracycline group and CMF group respectively (Khi^2^-test: p = 0.009). The rate of grade II and grade III skin dermatitis toxicity was 4.5% in the group A and 0% in the group B (Khi^2^-test: p = 0.013).

**Conclusion:**

From the 5 years retrospective investigation we showed similar disease free survival and overall survival in the two concurrent chemo-radiotherapy treatments based on anthracycline and CMF. However in the loco-regional breast cancer the treatment based on anthracycline was significantly better than that of the treatment based on CMF. There was more haematological and skin dermatitis toxicity in the anthracycline group.

## Background

In the case of early breast cancer and after radical mastectomy or conservative surgery, adjuvant radiotherapy is mandatory for diminishing the risk of recurrence [1-9]. Adjuvant chemotherapy is equally mandatory for diminishing metastasis recurrences [10-12]. However, the optimal sequence of treatments is not clearly defined and remains controversial. Several trials have shown that the incidence of spared metastasis is more important in the case of delay of chemotherapy, and local's recurrences are more frequents in the case of delay of radiotherapy [13,14]. Current standard treatment sequence is chemotherapy followed by radiotherapy. We are trying by this retrospective study to document and support the feasibility and efficiency of concurrent chemo-radiotherapy.

## Methods

### Patient selection

From January 2001 to December 2002, a large group of 244 patients with early breast carcinoma were selected at the National Institute of Oncology in Rabat, for investigation during treatment and up to now follow up. The patients were divided in two groups on the basis of chemotherapy treatment. In group A the treatment was based on anthracycline and in group B the treatment was based on CMF. Eighty four percent of the investigated cases (81% in group A and 86.5% in group B; Pearson-Khi^2 ^test: *p *= 0.23) had radical surgery [201 received Patey mastectomy and 4 received Halsted mastectomy (2 in group A and 2 in group B)] and the remaining 16% of the cases (19% in group A and 13.5% in group B; Pearson-Khi^2 ^test: *p *= 0.23) had breast conservative surgery [34 received tumorectomy and 5 received quadrentectomy (3 in group A and 2 in group B)]. All the 244 patients underwent concurrent adjuvant chemo-radiotherapy. In the concurrent chemo-radiotherapy both chemotherapy and radiotherapy were delivered at the same time. The median number of chemotherapy cycles delivered with radiotherapy was 2 (ranging from 1 to 5). Eighty percent of the patients (195 patients) received 2 or more chemotherapy cycles with concomitant radiotherapy. Patient medical records were retrospectively analysed and the following parameters were considered: demographic data, clinical stages, histological findings, treatment and outcome. Radiological, pathological and surgical reports were reviewed to determine the stage of the disease at the time of surgery by using the 2002 TNM classification for breast cancer [15]. The diagnostic instrumental examinations used to stage patients were: chest radiograph performed in all patients; abdominal ultrasound performed in all patients; and bone scan performed in only 16% of the patients (39 patients).

### Treatment plan

Data about treatment, notably surgery, chemotherapy and radiotherapy, were extracted from patient medical records. The date and site of recurrence and, if applicable, the date of death were also considered. The first group A of 110 patients was treated with anthracycline based protocol and the second group B of 134 patients was treated with CMF protocol. Additional file 1 and Diagram 1 summarizes the therapeutic strategy. According to the protocol followed at our institute, 95.5% of the patients received a radiotherapy treatment delivered to the whole breast or to thoracic wall (99.1% in group A and 92.5% in group B); in addition, the same 95.5% of the patients received a radiotherapy treatment delivered to the regional lymph nodes. The 4.5% of patients left received a radiotherapy treatment delivered to the whole breast or to the thoracic wall, in addition to a radiotherapy treatment delivered in the regional lymph nodes. All patients were treated with external beam radiotherapy using tangential fields of Co-60-gamma-Ray. The total delivered dose was 50 Gy, divided as 2-Gy daily fractions. The complementary treatment was given by electrons or by breast brachytherapy. The total complementary dose ranged from 10 to 20 Gy for 10 patients. Chemotherapy consisted of: a- intravenous CMF (cyclophosphamide 500 mg/m^2^, methotrexate 60 mg/m^2^, and 5-fluorouracil 500 mg/m^2^) on day 1, repeated every 21 days for six courses for 134 patients, b- intravenous AC60 (doxorubicin 60 mg/m^2 ^and cyclophosphamide 600 mg/m^2^) on day 1, repeated every 21 days for six courses for 57 patients, c- intravenous FEC75 (5-fluorouracile 500 mg/m^2^, epirubicin 75 mg/m^2^, and cyclophosphamide 500 mg/m^2^) on day 1, repeated every 21 days for six courses for 23 patients and d- intravenous FAC50 (5-fluorouracile 500 mg/m^2^, doxorubicin 50 mg/m^2^, and cyclophosphamide 500 mg/m^2^) on day 1, repeated every 21 days for six courses for 20 patients, and e- sequential treatment, repeated every 21 days for six courses for 10 patients (table 1, additional file 1 and diagram 1). We retrospectively compared toxicity, disease free survival and overall survival between two therapeutic groups A and B and between the sub-groups within A and B.

**Table 1:** Sequential treatments

**Protocol**	**Number of patients**
2AC60 → 4CMF*	2
2FAC50 → 4CMF*	1
6CMF* → 4AT	1
3FAC50 → 3CMF*	2
4CMF → 2FEC75*	1
2AC60 → 4CMF*	1
2CMF* → 4 AC	1
4AC* → 2CMF	1

* = regimen delivered in concomitant with radiotherapy; CMF = cyclophosphamide 500 mg/m^2^, methotrexate 60 mg/m^2^, and 5-fluorouracil 500 mg/m^2^; AC60 = doxorubicin 60 mg and cyclophosphamide 600 mg/m^2^; FEC75 = -fluorouracile 500 mg/m^2^, epirubicin 75 mg/m^2^, and cyclophosphamide 500 mg/m^2^; FAC50 = 5-fluorouracile 500 mg/m^2^, doxorubicin 50 mg/m^2^, and cyclophosphamide 500 mg/m^2^; AT = doxorubicin 50 mg/m^2 ^and docetaxel 75 mg/m^2^

### Statistical analysis

Overall survival (OS) and disease free survival (DFS) were analyzed statistically in all patients. Time to recurrence was calculated from the date of surgery to the date of first documented relapse or to the date of last follow up. Overall survival was calculated from the date of histological diagnosis (Fine Needle Aspiration, biopsy, or surgery) to the date of death or to the date of last follow up. The Kaplan-Meier method was used to estimate the rates of DFS, loco-regional recurrence-free survival (LRFS) and OS. The log-rank test was used to evaluate the differences between the two groups A and B. The distribution homogeneity was analyzed with the Pearson chi^2^-test for both groups and for all subgroups. The distribution of patient characteristics was partly imbalanced. The influence on survival of several prognostic factors (age, lymph node involvement, tumour volume, tumour grade, receptor status, and treatment regime) was analyzed by Cox regression. Statistical evaluation was carried out using SPSS 13.0 statistical software.

## Results

### Patient characteristics

Between January 2001 and December 2002, 244 women were retrospectively evaluated. One hundred ten patients received concurrent chemo-radiotherapy with anthracycline based regimen and 134 patients received concurrent chemo-radiotherapy with CMF based regimen. The demographic, clinical, pathologic, and therapeutic characteristics of the two groups of patients were summarized in table 2. After the analysis of homogeneity characteristics of the two groups we found more women aged less than 40 years (Khi^2^-test: p = 0.039) and more lymph node involvement (Khi^2^-test: p = 0.001) in the anthracycline group than in group B (table 2). The progesterone receptor status was the only statistically different subgroup from the three most important anthracycline sub-groups (Table 3). The homogeneity between the groups of patients managed either with mastectomy or breast conservative therapy (BCT) was also studied and summarized in table 4. For all patients, the mean delay of chemotherapy after surgery was 6.9 weeks (ranging from 0.7 to 37.9 weeks). And the mean delay of radiotherapy after surgery was 12.4 weeks (ranging from 2.4 to 53.3 weeks). In the two groups A and B respectively, 96.4% and 97.7% of the patients received the 6 courses of chemotherapy. All patients in the two groups received 100% of the planned radiotherapy dose.

**Table 2:** Demographic, clinical, histological, molecular and treatment characteristics of patients and analysis of groups homogeneity (test Pearson Khi^2^)

**Characteristic**	**Group A [n = 110] No (%)**	**Group B [n = 134] No (%)**	**p value**
**Age**			
<40	31 (28.2%)	23 (17.2%)	0.039
≥40	79 (71.8%)	111 (82.8%)	
**Menopausal status**			

No	75 (72.8%)	77 (62.1%)	0.087
Yes	28 (27.2%)	47 (37.9%)	
**Side**			

Right	48 (43.6%)	71 (53%)	0.146
Left	62 (56.4%)	63 (47%)	
**Surgery**			

Mastectomy (Patey or Halsted)	89 (80.9%)	116 (86.6%)	0.23
Conservative	21 (19.1%)	18 (13.4%)	
**Histology**			

DIC	99 (94,3%)	120 (90,9%)	0.33
LIC	6 (5.7%)	12 (9.1%)	
**SBR**			

I	6 (5.7%)	11 (8.5%)	0.567
II	69 (65.1%)	87 (66.9%)	
III	31 (29.2%)	32 (24.6%)	
**Hormonal receptors**			

**ER**			
Positive	77 (72%)	97 (73.5%)	0.793
Negative	30 (28%)	35 (26.5%)	
**PR**			
Positive	61 (57.5%)	82 (62.1%)	0.474
Negative	45 (42.5%)	50 (37.9%)	
**Tumour**			

pT1	16 (14.7%)	19 (14.5%)	0.732
pT2	62 (56.9%)	76 (58%)	
pT3	28 (25.7%)	29 (22.1%)	
pT4	3 (2.8%)	7 (5.3%)	
**pN, axillary**			

pN0	20 (18.2%)	40 (29.9%)	0.001
pN1	30 (27.3%)	42 (31.3%)	
pN2	31 (28.2%)	42 (31.3%)	
pN3	29 (26.4%)	10 (7.5%)	
**Breast/thoracic wall irradiation**			

Yes	109 (99.1%)	124 (92.5%)	0.014
No	1 (0.9%)	10 (7.5%)	
**Prophylactic supraclavicular fossa radiotherapy**			

Yes	104 (94.5%)	127 (94.8%)	0.936
No	6 (5.5%)	7 (5.2%)	
**Internal mammary radiotherapy**			

Yes	105 (95.5%)	128 (95.5%)	0.98
No	5 (4.5%)	6 (4.5%)	

**Axillary radiotherapy**			
Yes	18 (16.4%)	31 (23.1%)	0.189
No	92 (83.6%)	103 (76.9%)	

SBR = Scarf-Bloom-Richardson; DIC = ductal invasive carcinoma; LIC = lobular invasive carcinoma; ER = estrogen receptor; PR = progesterone receptor

**Table 3:** Analysis of anthracycline sub-groups (AC60, FEC75 and FAC50) homogeneity (test Pearson Khi^2^)

**Patients characteristics**	**Group FEC75 [n = 23] No (%)**	**Group FAC50 [n = 20] No (%)**	**Group AC60 [n = 57] No (%)**	**p value**
**Age**				

<40	8 (34.8%)	7 (35%)	14 (24.6%)	0.53
≥40	15 (65.2%)	13 (65%)	43 (75.4%)	
**Menopausal status**				

No	20 (90.9%)	14 (77.8%)	35 (66%)	0.075
Yes	2 (9.1%)	4 (22.2%)	18 (34%)	
**Side**				

Right	14 (60.9%)	7 (35%)	24 (42.1%)	0.188
Left	9 (39.1%)	13 (65%)	33 (57.9%)	
**Surgery**				

Mastectomy	5 (21.7%)	5 (25%)	8 (14%)	0.457
Conservative	18 (78.3%)	15 (75%)	49 (86%)	
**Histology**				

CCI	20 (95.2%)	19 (95%)	52 (94.5%)	0.992
CLI	1 (4.8%)	1 (5%)	3 (5.5%)	
**SBR**				

I	0	1 (5%)	4 (7.1%)	0.31
II	14 (70%)	10 (50%)	39 (69.6%)	
III	6 (30%)	9 (45%)	13 (23.2%)	
**Hormonal receptors**				

**ER**				
Positive	18 (81.8%)	11 (55%)	42 (76.4%)	0.106
Negative	4 (18.2%)	9 (45%)	13 (23.6%)	
**PR**				
Positive	17 (77.3%)	7 (35%)	31 (57.4%)	0.022
Negative	5 (22.7%)	13 (65%)	23 (42.6%)	
**Tumour**				

pT1	6 (26.1%)	1 (5%)	9 (16.1%)	0.514
pT2	10 (43.5%)	13 (65%)	32 (57.1%)	
pT3	7 (30.4%)	5 (25%)	13 (23.2%)	
pT4	0	1 (5%)	2 (3.6%)	
**pN, axillary**				

pN0	6 (26.1%)	3 (15%)	9 (15.8%)	0.807
pN1	4 (17.4%)	4 (20%)	15 (26.3%)	
pN2	7 (30.4%)	5 (25%)	18 (31.6%%)	
pN3	6 (26.1%)	8 (40%)	15 (26.3%%)	

FEC75 = 5-fluorouracile 500 mg/m^2^, epirubicin 75 mg/m^2^, and cyclophosphamide 500 mg/m^2^; FAC50 = 5-fluorouracile 500 mg/m^2^, doxorubicin 50 mg/m^2^, and cyclophosphamide 500 mg/m^2^; AC60 = doxorubicin 60 mg and cyclophosphamide 600 mg/m^2 ^; SBR = Scarf-Bloom-Richardson; DIC = ductal invasive carcinoma; LIC = lobular invasive carcinoma; ER = estrogen receptor; PR = progesterone receptor

**Table 4:** Analysis of demographic, clinical, histological, molecular and therapeutic characteristics of patients treated with mastectomy and breast conservative therapy (BCT) (test Pearson Khi^2^)

**Patients characteristics**	**Mastectomy [n = 205] No (%)**	**BCT [n = 39] No (%)**	**p value**
**Age**			

<40	48 (23.4%)	6 (15.4%)	0.268
≥40	157 (76.6%)	33 (84.6%)	
**Menopausal status**			

No	129 (67.5%)	23 (63.9%)	0.669
Yes	62 (32.5%)	13 (36.1%)	
**Side**			

Right	48 (43.6%)	71 (53%)	0.146
Left	62 (56.4%)	63 (47%)	
**Histology**			

DIC	183 (91.1%)	36 (100%)	0.062
LIC	18 (9%)	0	
**SBR**			

I	13 (6.6%)	4 (10.5%)	0.53
II	130 (65.7%)	26 (68.4%)	
III	55 (27.8%)	8 (21.1%)	
**Hormone receptors**			

**ER**			
Positive	120 (60.3%)	28 (71.8%)	0.877
Negative	79 (39.7%)	11 (28.2%)	
**PR**			
Positive	61 (57.5%)	23 (59%)	0.877
Negative	45 (42.5%)	16 (41%)	
**Tumour**			

pT1	25 (12.4%)	10 (25.6%)	0.003
pT2	111 (55.2%)	27 (69.2%)	
pT3	55 (27.4%)	2 (5.1%)	
pT4	10 (5%)	0	
**pN, axillary**			

pN0	49 (23.9%)	11 (28.2%)	0.285
pN1	57 (27.8%)	15 (38.5%)	
pN2	63 (30.7%)	10 (25.6%)	
pN3	36 (17.6%)	3 (7.7%)	
**Protocol**			

Anthracycline	89 (43.4%)	21 (53.8%)	0.23
CMF	116 (56.6%)	18 (46.2%)	
**Breast/thoracic wall irradiation**			

Yes	194 (94.6%)	39 (100%)	0.139
No	11 (5.4%)	0	
**Prophylactic supraclavicular fossa radiotherapy**			

Yes	193 (94.1%)	38 (97.4%)	0.402
No	12 (5.9%)	1 (2.6%)	
**Internal mammary radiotherapy**			

Yes	195 (94.1%)	38 (97.4%)	0.523
No	10 (5.9%)	1 (2.6%)	

**Axillary radiotherapy**			
Yes	42 (20.5%)	7 (17.9%)	0.717
No	163 (79.5%)	32 (82.1%)	

SBR = Scarf-Bloom-Richardson; DIC = ductal invasive carcinoma; LIC = lobular invasive carcinoma; ER = estrogen receptor; PR = progesterone receptor

### Treatment compliance

Analysis of haematological toxicity showed that the rate of grade III-IV neutropenia was 9.3% *vs *6.2% in group A and B respectively (Khi^2^-test: p = 0.4). The rate of grade II-III anaemia was 13.9% *vs *6.7% in anthracycline group and CMF group respectively (Khi^2^-test: p = 0.009) (Table 5). There was no cardiac toxicity that was clinically detectable in the two arms. The left ventricular fraction ejection (LVFE) was evaluated in only 7 patients (2 patients in the anthracycline group and 5 in the CMF group) and was normal (LVFE ranged between 63% and 87%). This constitutes the main limitation of our retrospective study. The second limitation was the skin dermatitis toxicity events which were noted in only few cases when the patients presented high toxicity grade. Therefore, we showed that 4.5% of the patients treated with anthracycline regimen had poor cosmetic results (grade II-III skin dermatitis toxicity), but in no patient of the group B the skin dermatitis toxicity was noted (Khi^2^-test: p = 0.013). The third limitation was the lake of pulmonary toxicity follow up in our data base. Nevertheless, only 2 patients in the AC60 sub-groups showed dry cough.

**Table 5:** Haematological toxicity

**Toxicity**	**Group A No (%)**	**Group B No (%) **	**p value**
Anemia			
Grade I	35 (32.4%)	25 (19.2%)	0.009
Grade II	13 (12%)	7 (5.4%)	
Grade III	2 (1.9%)	1 (1.3%)	
Grade IV	0		
Neutropenia			

Grade I	13 (12%)	15 (11.5%)	0.4
Grade II	27 (25%)	26 (20%)	
Grade III	8 (7.4%)	8 (6.2%)	
Grade IV	2 (1.9%)	0	
Thrombopenia			

Grade I	2 (1.9%)	2 (1.5%)	0.341
Grade II	2 (1.9%)	0	
Grade III	1 (0.9%)	0	
Grade IV	0	1 (0.8%)	

### Outcomes

**Figure 1:**
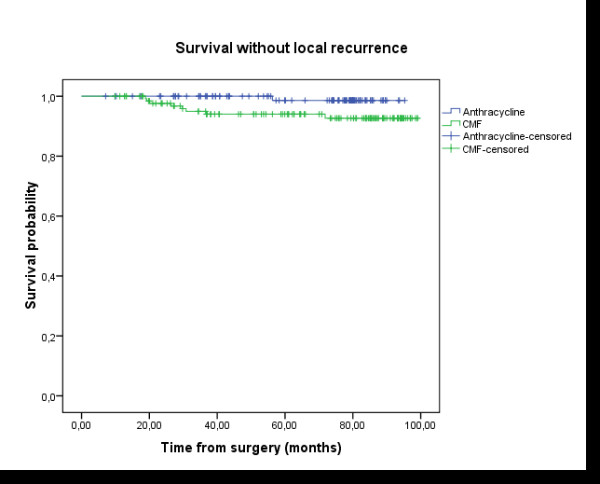
**Loco-regional-Free Survival (LRFS)**: the delay of LRFS was calculated by the date of surgery until the date of revealing of a loco-regional recurrence or until the date of death, or until the date of last news. The median follow-up, the rate of LRFS in five years, and the number of patients censored were presented. Group A (anthracycline): N = 110 (1 events, 109 censored); Group B (CMF): N = 134 (8 events, 126 censored); Survival probability at five years: 98.6% in group A vs 94% in group B; Log-rank test: p = 0.033.

**Figure 2:**
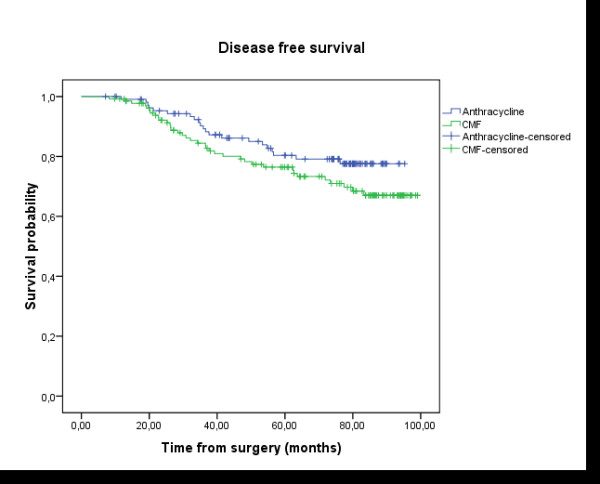
**Disease-Free Survival (DFS)**: the delay of DFS was calculated by the date of surgery until the date of revealing of a progress or until the date of death, or until the date of last news. The median follow-up, the rate of disease free survival in five years, and the number of patients censored were presented. Group A (anthracycline): N = 110 (21 events, 89 censored); Group B (CMF): N = 134 (36 events, 98 censored); Survival probability at five years: 80.4% in group A vs 76.4% in group B; Log-rank test: p = 0.136.

**Figure 3:**
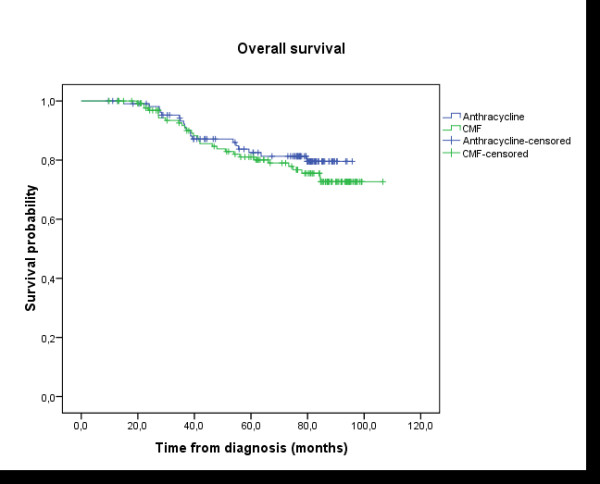
**Overall survival (OS)**: the delay of OS was calculated by the date of histological diagnosis until the death or until the date of last news. The median follow-up, the rate of overall survival in five years, and the number of patients censored were presented. Group A (anthracycline): N = 110 (19 events, 91 censored); Group B (CMF): N = 134 (29 events, 105 censored); Survival probability at five years: 82.5% in group A vs 81.1% in group B; Log-rank test: p = 0.428.

After 76.4 months median follow-up and 65.3 months mean follow up (ranging between 9.6 to 106 months), only one patient developed loco-regional relapse when the treatment was based on anthracycline. In contrast, 8 patients relapsed to loco- regional breast cancer in the CMF group. The 5 years loco-regional recurrence free survival rate was equal to 98.6% in group A *vs *94% in group B; Log-rank test: p = 0.033 (Figure 1). The 5 years rate of DFS was 80.4% in group A *vs *76.4% in group B; Log-rank test: p = 0.136 (Figure 2). The 5 years overall survival rate was 82.5% in group A vs 81.1% in group B; Log-rank test: p = 0.428 (Figure 3). Using univariate analysis, we found that the only prognosis factor influencing survival was the lymph node involvement status (p = 0.007) (Cox regression) (table 6).

**Table 6:** Univariate analysis of prognostic factors (Cox regression)

**Factor**	**P value**
Age: <40 vs ≥ 40	0.221
SBR: I vs SBR II-III	0.402
HR: positive vs negative	0.675
Tumour: pT1–2 vs pT3–4	0.263
Lymph node involvement: yes vs no	0.007
Regimen: CMF vs Anthracycline	0.428

**Figure 4:**
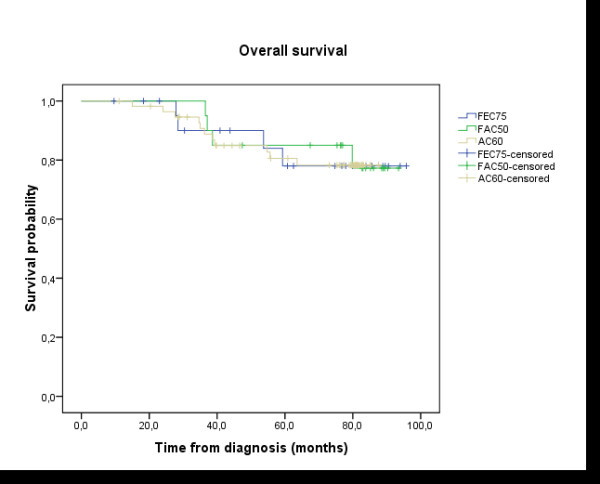
**Overall survival (OS)**: difference between the three anthracycline sub-groups: AC60, FEC75, and FAC50; Log-rank test: p = 0.982.

**Figure 5:**
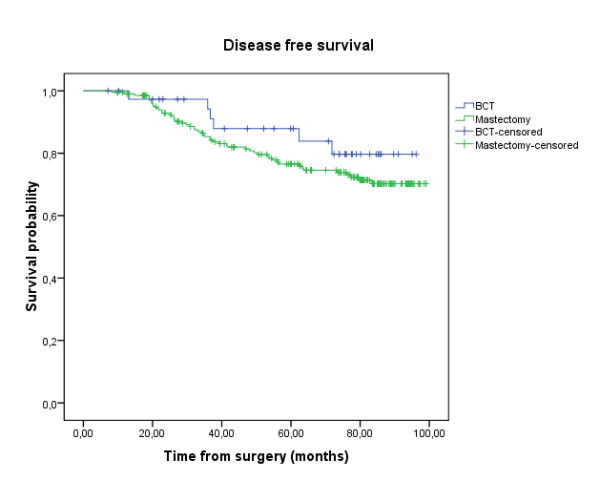
**Disease-Free Survival (DFS)**: mastectomy compared to breast conservative therapy; Log-rank test: p = 0.288.

**Figure 6:**
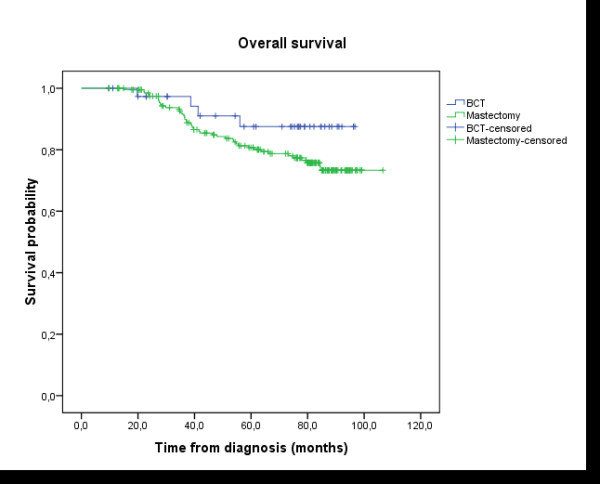
**Overall survival (OS)**: mastectomy compared to breast conservative therapy; Log-rank test: p = 0.173.

SBR = Scarf-Bloom-Richardson; HR = Hormonal receptors;

Analysis of the data showed no difference in survival between the 3 anthracycline cycles regimen: AC60, FEC75 and FAC 50; Log-rank test: p = 0.982 (Figure 4). And there was no difference in disease free survival and overall survival between the patients treated by mastectomy or breast conservative therapy (DFS: p = 0.288; OS: p = 0.173) (Figure 5 and 6).

## Discussion

Radiotherapy and chemotherapy after surgery are mandatory in the multidisciplinary management of early-stage breast cancer. Even if the optimal sequencing of theses treatments was largely studied during the last two decades, they remain controversial. Several retrospective studies have suggested an increase in local recurrence rates when radiotherapy was delivered after the end of chemotherapy treatment [13,14]. Hartsell et al showed that delays in the irradiation treatment were associated with increased risk of relapse in the breast cancer and recommended that radiotherapy treatment should be delivered within 120 days after breast surgery. Other authors showed that a delay in the initiation of RT for a period of 6 months or greater from diagnosis resulted in a higher local failure rate with an increased rate of distant metastases and a decreased overall survival rate. The Joint Centre for Radiation Therapy Trial (JCRT) confirmed theses results (rate of local recurrences was 5% vs 14% when radiotherapy was delayed) and suggested that radiotherapy should be delivered before chemotherapy [16]. However, other retrospective studies have suggested an increased rate of distant recurrences when RT was delivered before chemotherapy [17-20].

The current standard of care of early breast cancer was the surgery followed by chemotherapy followed by radiotherapy. Concurrent chemo-radiotherapy is a valuable method because of two advantages: 1. delivering the booths treatment in same time without any delay of chemotherapy or radiotherapy; 2. adjunction of chemotherapy to radiotherapy might produce a biological synergy effect that can increase the efficacy of the treatment [21]. Chemotherapy treatments based on liposomal doxorubicin, paclitaxel and vinorelbine, with concomitant RT in non operable and recurrent disease, were found to be of good efficacy and tolerability [21,22]. Reirradiation with concomitant chemotherapy was shown to have positive effect [21,23].

The promising results of concurrent chemo-radiotherapy showed in previous studies leaded us to investigate the efficacy and tolerability of this treatment in early breast cancer.

The objective of our contribution was to document and support the feasibility of concomitant treatment used at the national institute of oncology in Rabat and to confront our results to the results of 3 randomised studies published previously. Our work concerned the study of a data base of 244 patients treated by radical mastectomy (84%) or by BCT (16%) to compare efficacy and tolerability of two concomitant protocols: the first with anthracycline based regimen and the second with CMF regimen. After 76.4 months median follow-up and 65.3 months mean follow up (ranging between 9.6 to 106 months) we found no statistical difference in the DFS and the OS between the two therapeutic groups A and B. 5 years rate of DFS was 80.4% in group A *vs *76.4% in group B; Log-rank test: p = 0.136 and the 5 year overall survival rate was 82.5% in group A vs 81.1% in group B; Log-rank test: p = 0.428. However, to better explain these results and demonstrate the beneficial effect of one of the two protocols (anthracycline regimen and CMF regimen) over the other, the homogeneity of two groups was analysed. This analysis showed the presence of poorer prognosis factors in the anthracycline group (younger women and more lymph node involvement). In fact, there were significantly younger women (Pearson-Khi^2 ^test: p = 0.039) and more positive lymph nodes (Pearson-Khi^2 ^test: p = 0.001) in the anthracycline group. In addition, we showed significantly better local control in the anthracycline group; Log-rank test: p = 0.033. Overall, the patients in the two groups showed a very good loco-regional control.

In Europe, three recent randomised phase III trials were conducted to compare the sequential protocol (chemotherapy first) to the concomitant protocol: 1- in the first trial, 716 early breast cancers patients were treated by BCT and randomised into tow groups (ACROSEIN study) [24]. In the first group, the patients were treated by the FNC protocol (5-fluoro-uracil 500 mg/m2, mitoxantrone 12 mg/m^2 ^and cyclophosphamide 500 mg/m^2^) with concomitant radiotherapy. In the second group, the patients were treated by the FNC protocol followed by radiotherapy. The results showed no significant difference in both treatments for the 5-years DFS, LRFS, metastatic free survival, and OS. The two other studies [25,26] compared concurrent and sequential chemotherapy and radiotherapy after surgery for a reduced number of patients. In Italy, Arcangely et al [25] followed 206 patients that were randomly assigned to concurrent or sequential treatments. The two protocols were performed after quadrantectomy and axillary dissection for breast cancer with adjuvant chemotherapy (cyclophosphamide, methotrexate, and fluorouracil [CMF]). No significant differences were found in 5-years breast recurrence-free, metastasis-free, disease-free, and overall survival for the two groups of patients. In the third trial, Rouessé et al [26] followed 638 patients with prior breast surgery and positive axillary dissection (from which 416 were breast conservative surgery) and were randomly assigned to receive concomitant radiotherapy and chemotherapy (FNC protocol) or chemotherapy (fluorouracil, epirubicin, and cyclophosphamide protocol) followed by RT. No differences in 5-years disease-free and overall survival were observed in the two treatment groups. Nevertheless, in the ACROSEIN study the authors identified a significant decrease in the risk of loco-regional recurrence by 39% with concurrent radiotherapy and chemotherapy for node-positive patients. Rouessé et al [26] showed that concurrent treatment has a significantly better locoregional control in node-positive breast cancer after conservative surgery. In our study we found very good loco-regional control of the disease with only one loco-regional recurrence in the anthracycline goup and 8 in CMF group (p = 0.033). The main limitation of the tree European trials was the use of CMF protocol and FNC protocol without the use of anthracyclines and taxanes in the chemotherapy treatment. To our knowledge, our study is the first investigation which tests the efficacy and tolerability of the concomitant association of anthracycline regimen with radiotherapy. Our results confirm the superiority of this treatment to CMF regimen in term of local control. Anthracycline administered after RT showed a high incidence of severe skin dermatitis and oesophagitis, as reported by Recht et al [17]. In contrary to mitoxantrone, the anthracycline chemotherapy induces free-radical production that may potentiate normal tissue reactions. In ACROSEIN study, acute loco regional toxicities were moderate in the concomitant arm. Rouessé et al [26] presented more frequent grade 2 skin toxicities in the concomitant arm, and more sub clinical left ventricular ejection fraction events at 1 year (p = 0.02). In our study we showed more haematological toxicity when the treatment is based on anthracycline with significantly more grade II-III anaemia (13.9% vs 6.7%; Khi^2 ^test p = 0.009). Grade III-IV neutropenia (9.3% *vs *6.2%) and thrombopenia (0.9% vs 0.8%) were equally more frequent in anthracycline group but the differences were not significant. The lack of cardiac toxicity evaluation constitutes the main limitation of our retrospective study. Other limitations were the lack of skin and pulmonary toxicities evaluations. However, we can conclude that there was no clinical cardiac toxicity in the two groups and only 4.5% of the patients had poor cosmetic results in the anthracycline group versus 0% in the CMF group (Khi^2 ^test: p = 0.039).

## Conclusion

Concurrent chemo-radiotherapy is a valuable treatment protocol which shows promising results with good tolerability in non operable and recurrent breast cancer.

In early breast cancer, the previous published studies failed to show superiority of concurrent chemo-radiotherapy in term of survival.

From the present five years retrospective investigation we showed a similarity of the concurrent chemo-radiotherapy treatment results in DFS and OS and we identified a very good loco-regional control when this treatment was based on anthracycline.

Waiting for the results of ongoing research the standard of care is the use adjuvant chemotherapy prior to radiotherapy.

## Abbreviations

CMF: cyclophosphamide 500 mg/m^2^, methotrexate 60 mg/m^2^, and 5-fluorouracil 500 mg/m^2^; DFS: disease free survival; OS: overall survival; LRFS: loco-regional recurrence free survival; AC60: doxorubicin 60 mg/m^2 ^and cyclophosphamide 600 mg/m^2^; FEC75: 5-fluorouracile 500 mg/m^2^, epirubicin 75 mg/m^2^, and cyclophosphamide 500 mg/m^2^; FAC50: 5-fluorouracile 500 mg/m^2^, doxorubicin 50 mg/m^2^, and cyclophosphamide 500 mg/m^2^; BCT: breast conservative therapy; LVFE: left ventricular fraction ejection. FNC: 5-fluoro-uracil 500 mg/m2, mitoxantrone 12 mg/m2 and cyclophosphamide 500 mg/m2.

## Competing interests

The authors declare that they have no competing interests.

## Authors' contributions

NI: conception and design, acquisition of data, analysis and interpretation of data, statistical analysis, literature review, drafting the manuscript and revising it critically for important intellectual content; NM: acquisition and analysis of data; OM: acquisition and analysis of data; SE: acquisition and analysis of data; SA: acquisition and analysis of data; IB: acquisition and analysis of data; SA: acquisition and analysis of data; ZB: drafting the discussion; MAT: statistical analysis; AE: acquisition of data; OE: review of finale manuscript; NB: review of finale manuscript; BEKEG: review of finale manuscript; MI: involved in drafting the manuscript and revising it critically for important intellectual content; SA: statistical analysis; HE: conception, design and review of final manuscript.

## Supplementary Material

Additional file 1 **Diagram**. Diagram to summarize therapeutic strategy. Click here for file
