# Supplementary material for: Concurrent chemoradiotherapy in adjuvant treatment of breast cancer
Source: Radiat Oncol. 2009 Apr 7;4:12. doi: 10.1186/1748-717X-4-12 (PMC2679760; doi:10.1186/1748-717X-4-12)
Supplement: Additional file 1 — Diagram. Diagram to summarize therapeutic strategy. [file 1748-717X-4-12-S1.doc]

**Diagram 1:** Diagram summarize the therapeutic strategy

**244 patients treated between 2001 and 2002**

**Chirurgical treatment**

Group A: Mastectomy (81%) or conservative surgery (19%)

Group B: Mastectomy (86.5%) or conservative surgery (13.5 %)

**Adjuvant chemotherapy** (6 courses: cycles repeated every 21 days)

**CMF group**

(n = 134)

**Anthracycine group**

(n = 110)

**AC60**

(n = 57)

**FEC75**

(n = 23)

**Sequential treatment** (n = 10)

(n = 28)

Concurrent radiotherapy

**FAC50**

(n = 20)
